# P-1574. Asymptomatic Bacteriuria Not a Predictor of Clinical Failure in Uncomplicated Urinary Tract Infections (uUTI): A Prospective Analysis of Women Treated for uUTI from the REASSURE Trial

**DOI:** 10.1093/ofid/ofae631.1741

**Published:** 2025-01-29

**Authors:** Steven I Aronin, Michael Dunne, Sailaja Puttagunta

**Affiliations:** Iterum Therapeutics, Old Saybrook, CT; Bill & Melinda Gates Medical Research Institute, Old Saybrook, Connecticut; Iterum Therapeutics, Old Saybrook, CT

## Abstract

**Background:**

Per FDA *Guidance*, the primary efficacy endpoint for trials evaluating antimicrobials for treatment of UTI is a combined clinical/microbiologic response. Overall success requires both resolution of UTI symptoms and demonstration that the causative uropathogen is reduced to < 10^3^ CFU/mL at a fixed time point after randomization, regardless of whether the patient is asymptomatic. Previously, we retrospectively evaluated the impact of post treatment asymptomatic bacteriuria (ASB) for Phase 3 clinical trial patients with UTI and found that ASB was not a predictor of subsequent clinical failure. In this study, we prospectively assessed the impact of post treatment ASB on subsequent clinical response for adult women with uUTI.

**Figure:**
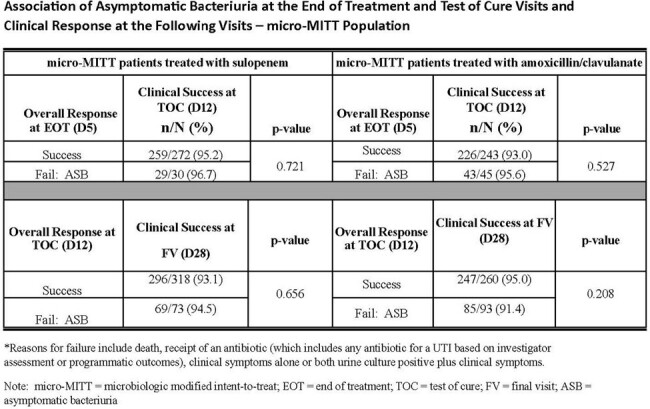

**Methods:**

Study IT001-310 was a Phase 3 randomized, double-blind, double-dummy, active controlled trial to evaluate the safety and efficacy of sulopenem/probenecid (SUL) versus amoxicillin/clavulanate (AMC) for the treatment of uncomplicated UTI (uUTI). Adult women were randomized to receive SUL or AMC, both bid for 5 days. The primary efficacy outcome was overall success (combined clinical/microbiologic success) at the Test of Cure (TOC) visit in the mMITT population. ASB at TOC was prespecified as an additional efficacy endpoint to be assessed, and the presence of ASB at the End of Treatment (EOT) and TOC visit was evaluated to see if it impacted clinical response at the following visit.

**Results:**

2,222 women were randomized; 990 (44.6%) were in the mMITT population. ASB was the reason for nonresponse in 74 (14.2%) and 93 (19.9%) patients treated with SUL and AMC, respectively. As shown in the table, for both treatment arms, the presence of ASB at the EOT and TOC visit did not lead to clinical failure at the following visit.

**Conclusion:**

SUL and AMC, both β-lactams, appear to have similar effects on the frequency of post treatment ASB. For patients in both treatment arms, the presence of ASB a week after completing UTI therapy was not a marker of subsequent clinical failure. The inclusion of ASB as part of the primary endpoint for studies of UTI should be reconsidered. Inclusion of microbiologic results from asymptomatic patients after complete resolution of uUTI symptoms is a practice inconsistent with available treatment recommendations.

**Disclosures:**

**Steven I. Aronin, MD**, Iterum Therapeutics: Employee|Iterum Therapeutics: Employee|Iterum Therapeutics: Stocks/Bonds (Public Company)|Iterum Therapeutics: Stocks/Bonds (Public Company) **Michael Dunne, MD**, Iterum Therapeutics: Advisor/Consultant|Iterum Therapeutics: Board Member|Iterum Therapeutics: Stocks/Bonds (Public Company) **Sailaja Puttagunta, MD**, Iterum Therapeutics: Employee|Iterum Therapeutics: Employee|Iterum Therapeutics: Stocks/Bonds (Public Company)|Iterum Therapeutics: Stocks/Bonds (Public Company)

